# LCN2 regulates the gut microbiota and metabolic profile in mice infected with *Mycobacterium bovis*

**DOI:** 10.1128/msystems.00501-24

**Published:** 2024-07-25

**Authors:** Quntao Huang, Junhong Xing, Guoli Li, Mengting Liu, Mengtian Gao, Jingwen Wang, Fang Tang, Jianluan Ren, Chengzhu Zhao, Xinru Wang, Xinyu Zhou, Haodong Luo, Youli Yu, Dexin Zeng, Jianjun Dai, Feng Xue

**Affiliations:** 1 MOE Joint International Research Laboratory of Animal Health and Food Safety, Key Laboratory of Animal Bacteriology, Ministry of Agriculture, College of Veterinary Medicine, Nanjing Agricultural University, Nanjing, China; 2 College of Veterinary Medicine, Jilin Provincial Engineering Research Center of Animal Probiotics, Jilin Provincial Key Laboratory of Animal Microecology and Healthy Breeding, Engineering Research Center of Microecological Vaccines (Drugs) for Major Animal Diseases, Ministry of Education, Jilin Agricultural University, Changchun, China; 3 Department of Chronic Communicable Disease, Center for Disease Control and Prevention of Jiangsu Province, Nanjing, China; 4 Institute of Animal Science, Ningxia Academy of Agriculture and Forestry Sciences, Yinchuan, China; 5 Technology Center of Hefei Customs, and Anhui Province Key Laboratory of Analysis and Detection for Food Safety, Hefei, China; 6 College of Pharmacy, China Pharmaceutical University, Nanjing, China; University of California, San Diego, La Jolla, California, USA

**Keywords:** Lcn2, *M. bovis*, gut microbiota, non-targeted metabolome

## Abstract

Infection with *Mycobacterium bovis* precipitates a spectrum of pathologies in bovines, notably necrotic pneumonia, mastitis, and arthritis, impinging upon the health and nutritional assimilation of these animals. A pivotal factor, lipocalin 2 (Lcn2), is responsive to microbial invasion, inflammatory processes, and tissue damage, the extent of which Lcn2 modulates the gut environment, however, remains unclear in response to *M. bovis*-induced alterations. To explore the role of Lcn2 in shaping the gut milieu of mice during a 5-week period post*-M. bovis* infection, Lcn2 knockout Lcn2^−/−^ mice were scrutinized for changes in the gut microbiota and metabolomic profiles. Results showed that Lcn2^−/−^ mice infected with *M. bovis* exhibited notable shifts in the operational taxonomic units (OTUs) of gut microbiota, alongside significant disparities in α and β diversity. Concomitantly, a marked increase was observed during the 5-week period in the abundance of Akkermansia, Oscillospira, and Bacteroides, coupled with a substantial decrease in Ruminococcus within the microbiome of Lcn2 knockout mice. Notably, *Akkermansia muciniphila* was significantly enriched in the gut flora of Lcn2^−/−^ mice. Furthermore, the absence of Lcn2 significantly altered the gut metabolomic landscape, evidenced by elevated levels of metabolites such as taurodeoxycholic acid, 10-undecenoic acid, azelaic acid, and dodecanedioic acid in Lcn2^−/−^ mice. Our findings demonstrated that the lack of Lcn2 in the context of *M. bovis* infection profoundly affected the regulation of gut microbiota and metabolomic components, culminating in a transformed gut environment. Our results revealed that Lcn2 may regulate gut microbiota and metabolome components, changing the intestinal environment, thereby affecting the infection status of *M. bovis*.

**IMPORTANCE:**

Our study addresses the critical knowledge gap regarding the specific influence of lipocalin 2 (LCN2) in the context of *Mycobacterium bovis* infection, particularly focusing on its role in the gut environment. Utilizing LCN2 knockout (Lcn2^−/−^) mice, we meticulously assessed changes in the gut microbiota and metabolic components following *M. bovis* infection. Our findings reveal alterations in the gut microbial community, emphasizing the potentially crucial role of LCN2 in maintaining stability. Furthermore, we observed significant shifts in specific microbial communities, including the enrichment of *Akkermansia muciniphila*, known for its positive impact on intestinal health and immune regulation. The implications of our study extend beyond understanding the dynamics of the gut microbiome, offering insights into the potential therapeutic strategies for gut-related health conditions and microbial dysbiosis.

## INTRODUCTION

*Mycobacterium bovis* is a significant zoonotic pathogen that primarily causes tuberculosis (TB) in cattle and various other domesticated and wild animals (1). It shares many pathological and clinical characteristics with *Mycobacterium tuberculosis*, the primary cause of TB in humans (2). *M. bovis* accounts for a portion of human TB cases, especially in regions where humans and cattle live in close proximity (3). Historically, before the widespread adoption of pasteurization, *M. bovis* was a more common cause of TB in humans as it could be transmitted through the consumption of raw or unpasteurized dairy products (4). The bacterium can also be transmitted through aerosol droplets, posing a concern in areas with substandard cattle management practices (5). Globally, *M. bovis* TB in cattle remains a significant issue in many countries, particularly in parts of Africa, Latin America, and Eastern Europe (6). The infection not only impacts animal health and welfare but also imposes a substantial economic burden due to livestock loss and control measures (7). Studying and controlling *M. bovis* is crucial not only for animal health but also for understanding and managing TB within a One Health context. This approach recognizes the interconnected health of people, animals, and their shared environment (8).

Lipocalin 2 (LCN2), a remarkably versatile molecule, undergoes induction in the central nervous system across a spectrum of acute and chronic pathological states (9). Also recognized as neutrophil gelatinase-associated lipocalin and siderocalin (Scn), this 25-kDa protein is intricately linked with inflammatory responses in various diseases (10). LCN2 plays a pivotal role in regulating iron homeostasis; although incapable of inherently binding iron, it exhibits an affinity for siderophores, a diverse group of high-affinity iron-chelating compounds (11). Currently, six receptors have been identified for LCN2, namely, neutrophil gelatinase-associated lipocalin receptor, low-density lipoprotein-related protein 2 (LRP2), LRP6, melanocortin 4 receptor (MC4R), MC1R, and MC3R (12). Furthermore, LCN2 secreted by neutrophils infiltrating tissues can induce ferroptosis and atrophy in adipose and muscle tissues of patients with cancer cachexia (13). Additionally, Lcn2 can mitigate the inflammatory damage caused by *Escherichia coli* O157:H7 infection in mice by enhancing the functionality of the intestinal barrier (14). The gut microbiome constitutes a pivotal element in human health, playing an indispensable role in digestion, immune system functionality, and disease prevention (15). These microorganisms not only facilitate the breakdown of nutrients in food but also actively contribute to the synthesis of vitamins and essential amino acids (16). Recent research has shed light on the intricate interplay between the gut microbiota and the host’s metabolic, immune, and nervous systems (17). Dysbiosis, marked by an imbalance of gut microbiota, has been implicated in the development of a myriad of diseases, including obesity, diabetes, cardiovascular diseases, and specific types of cancer (18). Furthermore, the gut microbiome’s influence extends to mental health, with alterations in the gut flora potentially contributing to the onset of depression and anxiety disorders (19). The gut microbiota exerts regulatory effects on the host’s metabolism through the production of metabolites, acting as paracrine or endocrine factors and influencing the metabolism of bile acids, amino acid derivatives, bioactive lipids, and specific neurotransmitters such as gamma-aminobutyric acid (GABA), serotonin, and nitric oxide (20). The intricate relationship between the gut microbiome and health is further underscored by metabolites like short-chain fatty acids, bile acids, and trimethylamine N-oxide, which impact the host through specific receptors, including PPARα, PPARγ, AhR, GPR41, GPR43, GPR119, and Takeda G protein-coupled receptor 5 (21).

In our study, we have uncovered the pivotal role of Lcn2 in orchestrating the regulation of gut microbiota and metabolic components in mice subjected to *M. bovis* infection. Additionally, we propose a potential interactive mechanism between the *Lcn2* gene and *Akkermansia muciniphila*, which could modulate the dynamics of *M. bovis* infection. These findings not only deepen our understanding of the biological functions of Lcn2 but also lay the groundwork for the development of novel therapeutic strategies targeting *M. bovis* infection.

## MATERIALS AND METHODS

### Animals

Six-week-old, female, specific pathogen-free (SPF) C57BL/6 mice were purchased from Yangzhou University (Comparative Medicine Center).

### Bacterial strains

Virulent *M. bovis* Beijing strain (#BD271310, BD Biosciences, USA) was acquired from the China Institute of Veterinary Drug Control and was cultured in 7H9 Middlebrook media containing 0.05% Tween-80 (#9005-65-6, Sigma-Aldrich, USA) and 10% albumin-dextrose-catalase (#211886, BD Biosciences, USA) enrichment solution. Growth was determined at 37°C for a 1-week to medium logarithmic period.

### *M. bovis* infection experiment

The mice were raised in a level III biosafety facility (Jiangsu Provincial Center for Disease Control and Prevention, Nanjing, China). The mice were divided into two groups of six mice each (control and Lcn2 knockout [KO]). In every group, 5 × 10^5^ colony-forming units (CFU) of *M. bovis* per mouse were given intranasally. After *M. bovis* infection, the feces were collected from two groups of mice once a week. The mice were sacrificed by neck dislocation under inhaled isoflurane anesthesia. The mice were sacrificed on days 30 and 45, samples were collected, spleen was prepared as a single-cell suspension for flow cytometry assay, blood was collected for enzyme-linked immunosorbent assay (ELISA) detection of inflammatory factors, and lungs were harvested for preparation of pathological sections for hematoxylin and eosin (H&E) staining.

### Lcn2^−/−^ mouse generation

The *Lcn2* gene (NCBI Reference Sequence: NM_008491.1; Ensembl: ENSMUSG00000026822) is located on mouse chromosome 2. Six exons were identified, with the ATG start codon in exon 1 and the TGA stop codon in exon 6 (Transcript Lcn2-201: ENSMUST00000050785). Exon 2 was selected as the target site. Cas9 and gRNA were co-injected into fertilized eggs for KO mouse production. The pups were genotyped by PCR followed by sequencing analysis. The KO region was approximately 707 bp, which does not have any other known gene. The primers for genomic DNA PCR-based genotyping were as follows: F1 (forward), 5′-CAACTCAGAACTTGATCCCTGCC-3′, and R1 (reverse), 5′-TTTCCCTAAGTCCCGTTCAATCC-3′.

### 16S rRNA gene sequencing and analysis

A sample was extracted using a genomic DNA purification kit from Sangon Biotech Co., Ltd. (Shanghai, China). Bacterial 16S rRNA sequencing genes (V3–V4 region) were amplified by primer pairs (341 F, 5′-CCTAYGGGRBGCASCAG-3′; 806R, 5′-GGACTACHVGGGTWTCTAAT-3′). PCR amplification products were detected by 2% agarose gel electrophoresis, and were recovered and purified using AxyPrep DNA kit (Axygen Biosciences, Union City, CA, USA). The purified PCR products were quantitatively detected by QuantiFluor-ST (Promega, USA), and the qualified sequencing library was sequenced by NovaSeq sequencer. Microbiome testing services were supported by Wekemo Tech Group Co., Ltd. (Shenzhen, China). The original fastq files were processed using QIIME 2.0, and representative sequences and feature tables were exported, followed by species annotation and visualization. Bacterial 16S rRNA was referenced to Greengenes database. Gene functional analysis was performed using the PICRUSt2 software package.

### Detection of non-target metabolites by LC-MS/MS

The sample extraction steps and instrument parameters were as described in a previous study . The sample (100 µL) was pipetted into an Eppendorf tube. Then, 200 µL of methanol (including 1 µg/mL internal standard) was added and vortexed to mix for 15 min. The extract was centrifuged at 20,000 × *g* for 10 min at 4°C, and the supernatant was collected, following which the supernatant was dried using vacuum drying. All extracts were dissolved in 200 µL of ice-cold methanol and then sonicated in an ice bath for 10 min to facilitate dispersion prior to liquid chromatograph mass spectrometer/mass spectrometer (LC-MS/MS analysis. LC-MS/MS analysis and metabolite profiling were also performed as previously described, which mainly was analyzed by open source software MSDIAL 4.0. Moreover, pathway analysis (enrichment analysis, pathway map) of characteristic metabolites was implemented by MetaboAnalyst 5.0.

### Flow cytometry

CD3,4,8 antibodies were used to stain single-cell suspensions of the spleen (SP). All antibodies were purchased from BD Biosciences (New York, NY, USA). The samples were incubated at 4°C in the dark for 30 min, then 200 µL of bdfacs lysis solution was added. The samples were again incubated for 10 min at room temperature and were centrifuged at 500 × *g* at 4°C. The cell pellet was resuspended in 200 µL of ice-cold cell buffer. BD fluorescence-activated cells were sorted and analyzed by FACS using an LSRFortessa analyzer (BD Biosciences, USA). All data were analyzed using FlowJo 7.6 software.

### Histology

Left lung lobes were fixed overnight with 4% paraformaldehyde and were transferred into 70% ethanol the following day until processing. The processed tissue were embedded in paraffin, and 4-µm sections were cut and stained with H&E.

### Enzyme-linked immunosorbent assay

The blood was centrifuged at 5,000 × *g* for 10 min to collect plasma. Cytokine levels of IL-6, IL-10, IL-12, TNF-α, and IFN-γ in plasma were evaluated by standard ELISA kits [Jiangsu Meimian Industrial Co. Ltd. (Jiangsu, China)] following the manufacturer’s guidelines.

### Statistical analysis

All data were analyzed with Graphpad Prism 8.0 software. Student’s *t*-test was used for pairwise comparisons, and one-way analysis of variance (ANOVA) was used for comparisons of more than two groups. The values are shown as the mean ± SEM. *P < 0.05, **P < 0.01, ***P < 0.001.

## RESULTS

### Lcn2 deletion aggravates *M. bovis* infection

**Fig 1:**
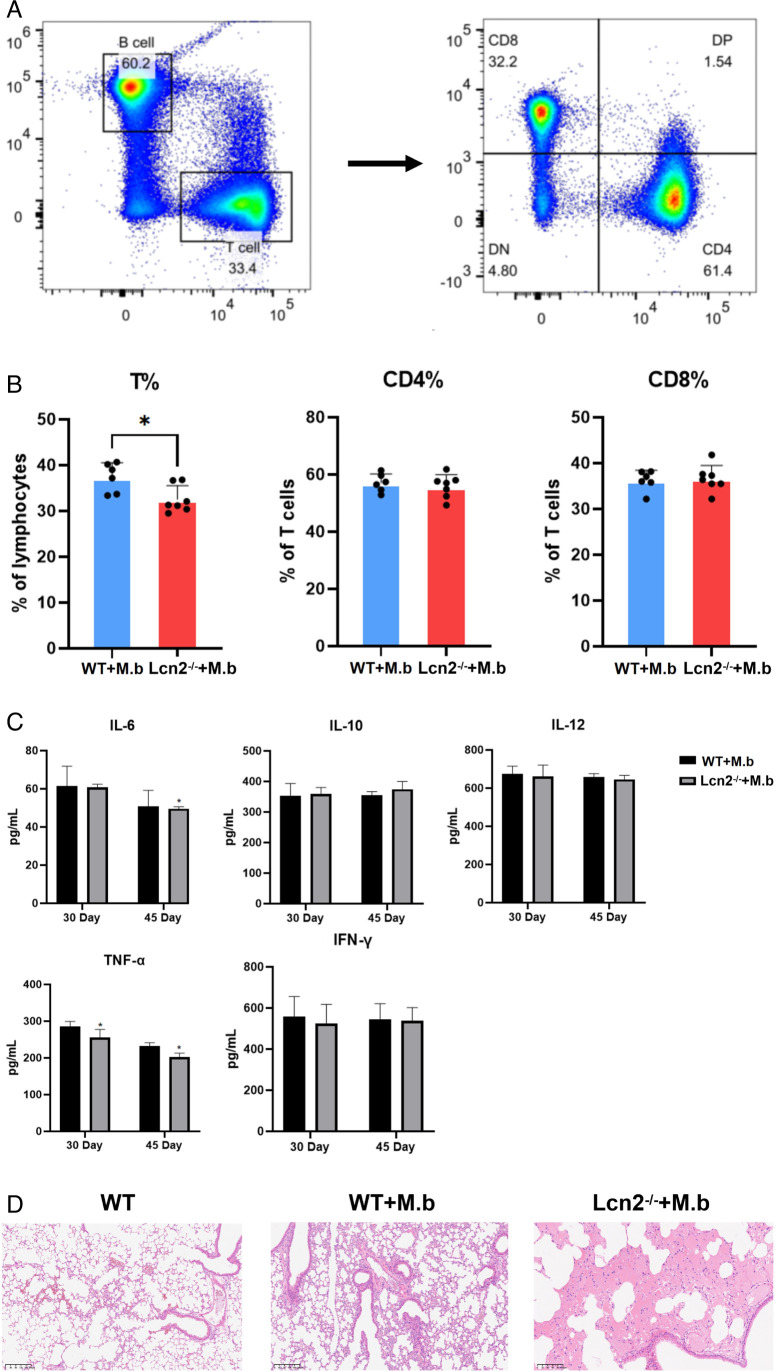
Differential analysis of *M. bovis* infection in WT and Lcn2^−/−^ mice. (**A**) Gating strategy for flow cytometry. (**B**) Quantification of CD3, CD4, and CD8^+^ T cells in the spleens of mice. (**C**) Analysis of IL-6, IL-10, IL-12, TNF-α, and IFN-γ cytokines. (**D**) Histological analysis of mouse lung tissue. The results are presented as the means ± SD, and statistical significance was calculated by *t*-tests for two groups. *P < 0.05.

To elucidate the role of Lcn2 in *M. bovis* infection, we established a murine model of *M. bovis* infection using wild-type (WT) and Lcn2-deficient mice. T cells can mitigate *M. bovis* infection by activating macrophages to enhance their bactericidal capacity and by exerting cytotoxic effects to kill target cells. Following *M. bovis* infection, we observed a significant decrease in T cells in Lcn2^−/−^ mice compared to wild-type mice (Fig. 1A and B). Previous studies have demonstrated the pro-inflammatory function of Lcn2; therefore, we assessed the levels of IL-6, IL-10, and IL-12 in this study. Interestingly, we found a significant downregulation of IL-6 and TNF-α levels in Lcn2^−/−^ mice (Fig. 1C). Furthermore, we analyzed the extent of lung damage in the mice. Our results revealed that Lcn2^−/−^ mice exhibited less severe lung damage compared to wild-type mice, characterized by moderate alveolar edema, mild alveolar dilation, and minimal perivascular lymphocyte infiltration (Fig. 1D). This result was consistent with the mental and physiological states of the mice, and the mental state of the Lcn2^−/−^ mice was significantly more depressed and the symptoms of diarrhea were more severe.

### Infection with *M. bovis* leads to alterations in the number of OTUs of gut microbiota in Lcn2^−/−^ mice

**Fig 2:**
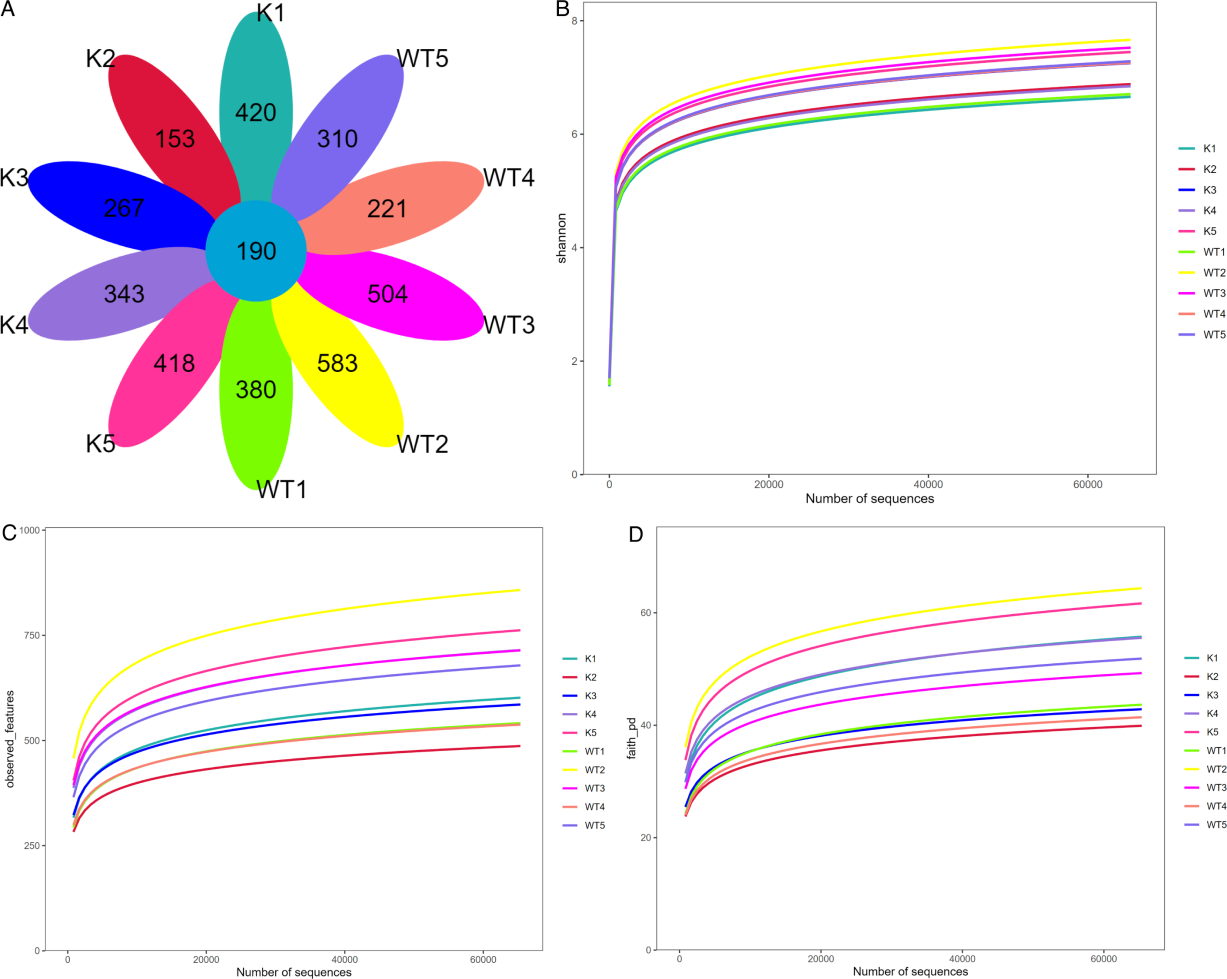
The gut microbiota analysis in WT and Lcn2^−/−^ mice included curves for the OTUs obtained from 30 samples. (**A**) Statistics on the number of common and unique OTUs. (**B**) Shannon-Wiener curves. (**C**) Observed species number. (**D**) Faith curves. K, samples from lcn2^−/−^ mice; WT, samples from wild-type mice.

16S rRNA sequencing was utilized to examine the changes in the gut microbiota of wild-type and Lcn2^−/−^ mice over a period of 1–5 wk postinfection. The operational taxonomic unit (OTU) analysis revealed a total of 190 shared OTUs. The unique counts for wild-type mice in weeks 1 to 5 were 380, 583, 504, 221, and 310, respectively. For Lcn2 knockout mice, the counts were 420, 453, 267, 343, and 418 (Fig. 2A). Additionally, assessments using the Shannon index (Fig. 2B), species observation counts (Fig. 2C), and Faith’s diversity index (Fig. 2D) indicated a uniform distribution of species, reasonable sequencing richness, and adequate sequencing depth.

### The gut microbiota diversity in Lcn2^−/−^ mice exhibits significant alterations

**Fig 3:**
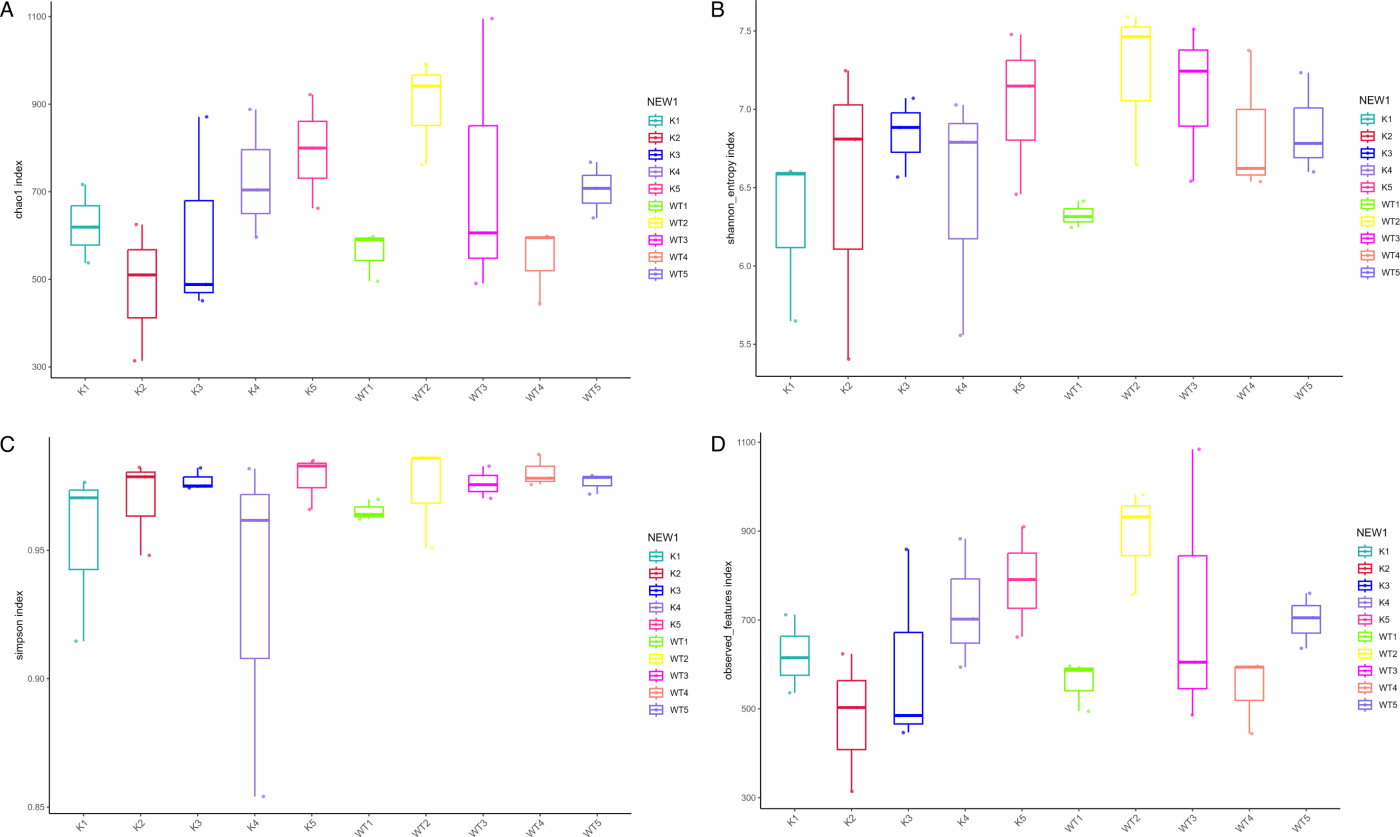
Analysis of gut microbiota α diversity in *M. bovis*-infected WT and Lcn2^−/−^ mice. Chao1 (**A**), Shannon index (**B**), Simpson index (**C**), and observed number of species (**D**) were used as α diversity estimators. K, samples from lcn2^−/−^ mice; WT, samples from wild-type mice.

**Fig 4:**
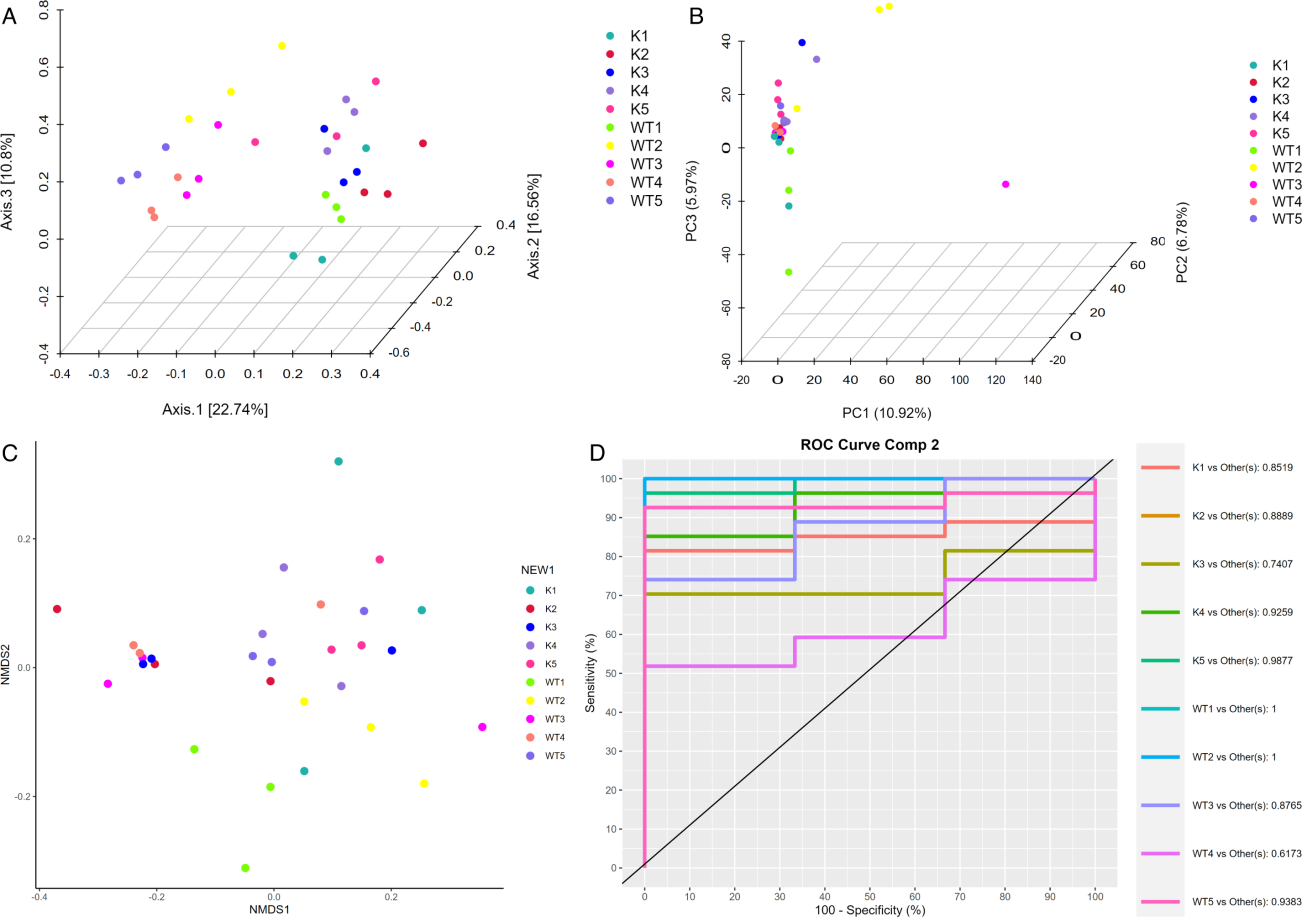
Analysis of gut microbiota β diversity in *M. bovis*-infected WT and Lcn2^−/−^ mice. PCoA (**A**), PCA (**B**), NMDS (**C**), and PLS-DA analysis (**D**) were used as β diversity estimators. K, samples from lcn2^−/−^ mice; WT, samples from wild-type mice.

To evaluate the diversity of gut microbiota in wild-type and knockout mice, we conducted an α diversity analysis. The Chao1 (Fig. 3A), Shannon (Fig. 3B), Simpson (Fig. 3C), and observed species indices (Fig. 3D) revealed notable differences between wild-type and Lcn2 knockout mice during the 1- to 5-week period following infection with the *M. tuberculosis* complex. Additionally, the gut microbiota of each mouse type displayed dynamic changes throughout the infection. We further explored the β diversity in both mouse types postinfection, characterized through principal coordinate analysis (PCoA) (Fig. 4A), principal component analysis (PCA) (Fig. 4B), nonmetric multidimensional scaling (NMDS) (Fig. 4C), and partial least-squares discriminant analysis (PLS-DA) (Fig. 4D) analyses. The results indicated that, in the infected state, the spatial structure of the gut microbiota in Lcn2 knockout mice underwent significant changes compared to wild-type mice, as evidenced by alterations in sample distances. This suggests that the gut microbiota of both groups is diverse and exhibits significant differences over time.

### The absence of Lcn2 regulates the composition of gut microbiota species

**Fig 5:**
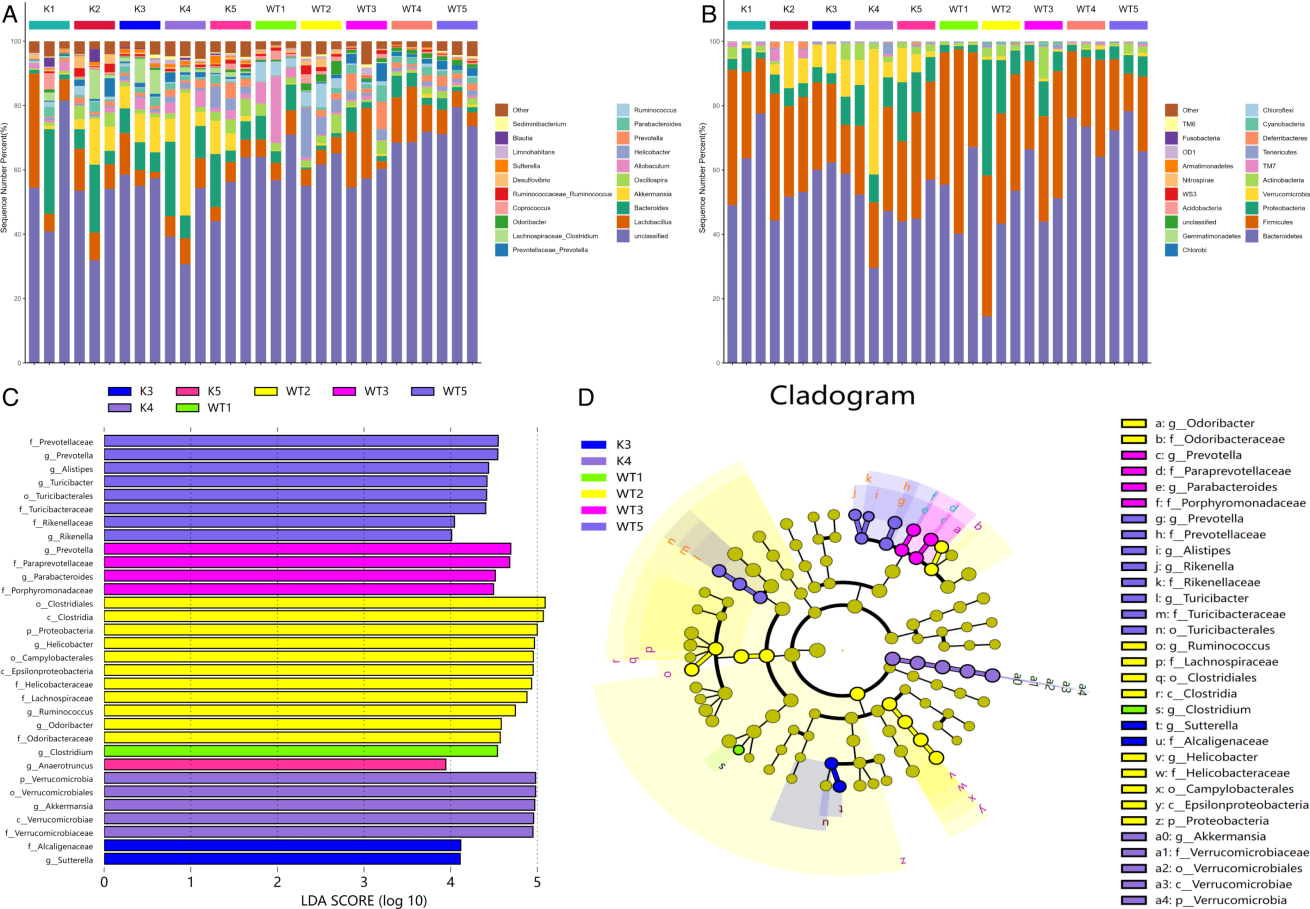
Species composition difference analysis. Species composition structure analysis of each group of samples at the phylum (**A**) and genus (**B**) levels. An LDA effect size of greater than 4 was used as a threshold for the LEfSe analysis (**C**). Cladogram of the LEfSe analysis of the gut microbiota in different groups (**D**). K, samples from lcn2^−/−^ mice; WT, samples from wild-type mice.

To characterize the species composition in both mouse types, we conducted a microbiota analysis at the phylum (Fig. 5A) and genus (Fig. 5B) levels. The results revealed notable differences. At the phylum level, there was a significant increase in the abundance of Verrucomicrobia in the Lcn2^−/−^ mice compared to wild-type mice during weeks 1–5. Moving to the genus level, the Lcn2 knockout mice exhibited a significant increase in the abundance of Akkermansia, Oscillospira, and Bacteroides during weeks 1–5, whereas the abundance of Ruminococcus decreased. Furthermore, employing the linear discriminant analysis effect size (LEfSe) method to identify significant biological markers, we observed distinct patterns in Lcn2^-/-^ mice after 3 wk of *M. bovis* infection. Specifically, the abundance of Verrucomicrobia, Verrucomicrobiales, Akkermansia, Verrucomicrobiae, Verrucomicrobiaceae, and Alcaligenaceae significantly increased. Notably, Sutterella’s abundance increased after 4 wk of infection. For WT mice, the abundance of Clostridium significantly increased in the first week postinfection, whereas that of Odoribacter, Ruminococcus, and Helicobacter significantly increased in the second week postinfection. Additionally, Prevotella’s abundance showed a significant increase in the third and fourth weeks postinfection (Fig. 5C and D).

### *A. muciniphila* is significantly enriched in the gut microbiota of Lcn2^−/−^ mice

**Fig 6:**
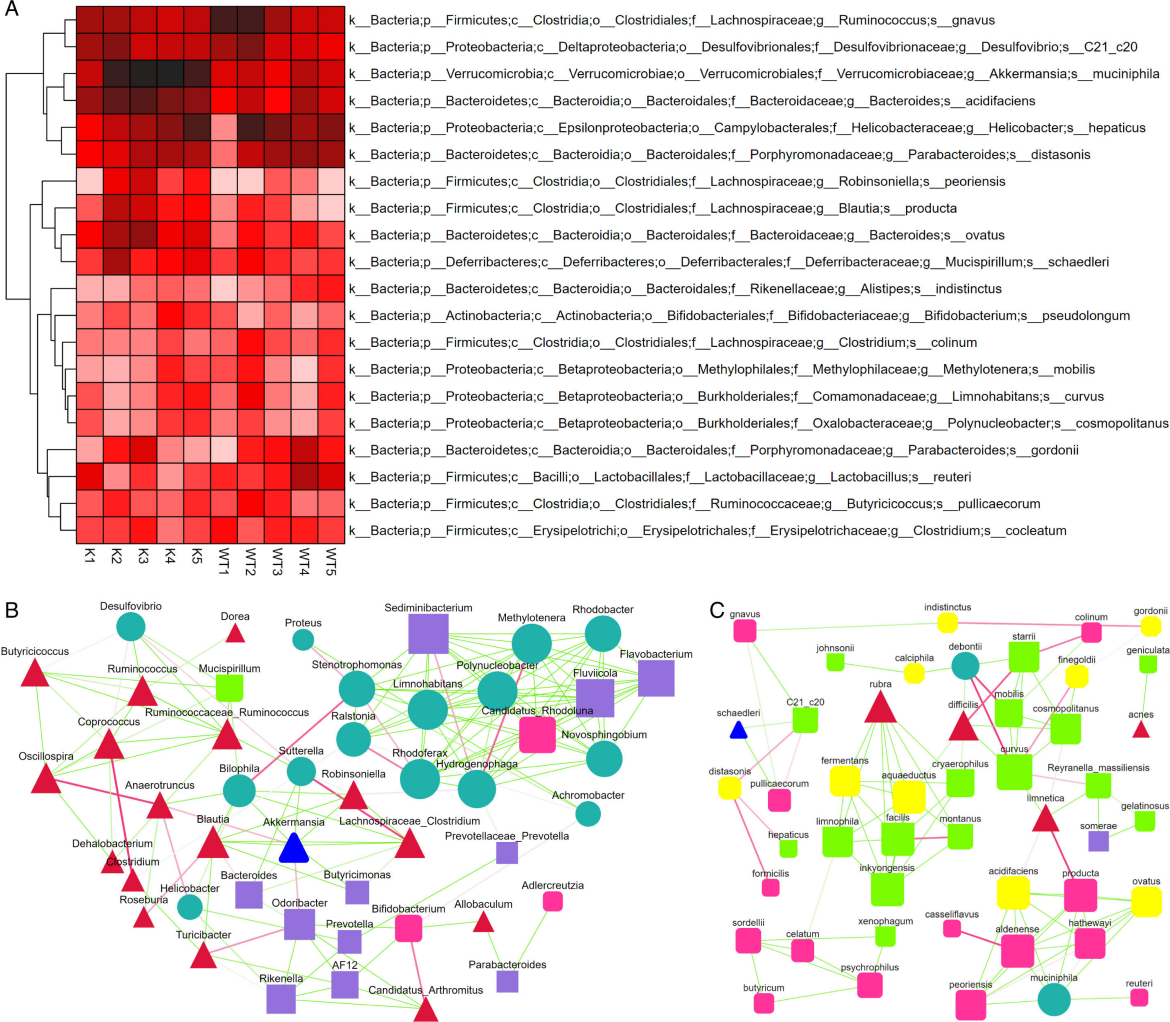
Gut microbial interaction analysis. Cluster analysis was performed on the samples to construct a heat map arranged in group order, and the similarities or differences between the analyzed groups were characterized (**A**). The correlation index (Pearson correlation coefficient) was calculated for all samples, and the bacterial genus (**B**) and strain (**C**) level to obtain the species correlation coefficient matrix. K, samples from lcn2^−/−^ mice; WT, samples from wild-type mice.

Microbial enrichment analysis post*-M. tuberculosis* infection unveiled a heightened enrichment of *A. muciniphila* in the gut microbiota of Lcn2^−/−^ mice compared to wild-type mice. Noteworthy findings from recent studies emphasize the pivotal role of *A. muciniphila* in regulating intestinal barrier function, maintaining gut microbiome balance, and modulating the host immune system. This suggests a potential association with the absence of the *Lcn2* gene (Fig. 6A). Subsequently, we delved into the interaction networks of the microbiota at both the genus and species levels in both mouse types. At the genus level, Akkermansia displayed interactions with several genera, including Bilophila, Sutterella, Blautia, Bacteroides, and Odoribacter (Fig. 6B). Zooming in at the species level, *A. muciniphila* exhibited interactions with various species such as *Lactobacillus reuteri*, Peoriensis, Aldenense, and Hathewayi. These findings underscore the significant contribution of *A. muciniphila* during the infection process of *M. tuberculosis* (Fig. 6C).

### *Lcn2* gene regulates intestinal metabolic components

**Fig 7:**
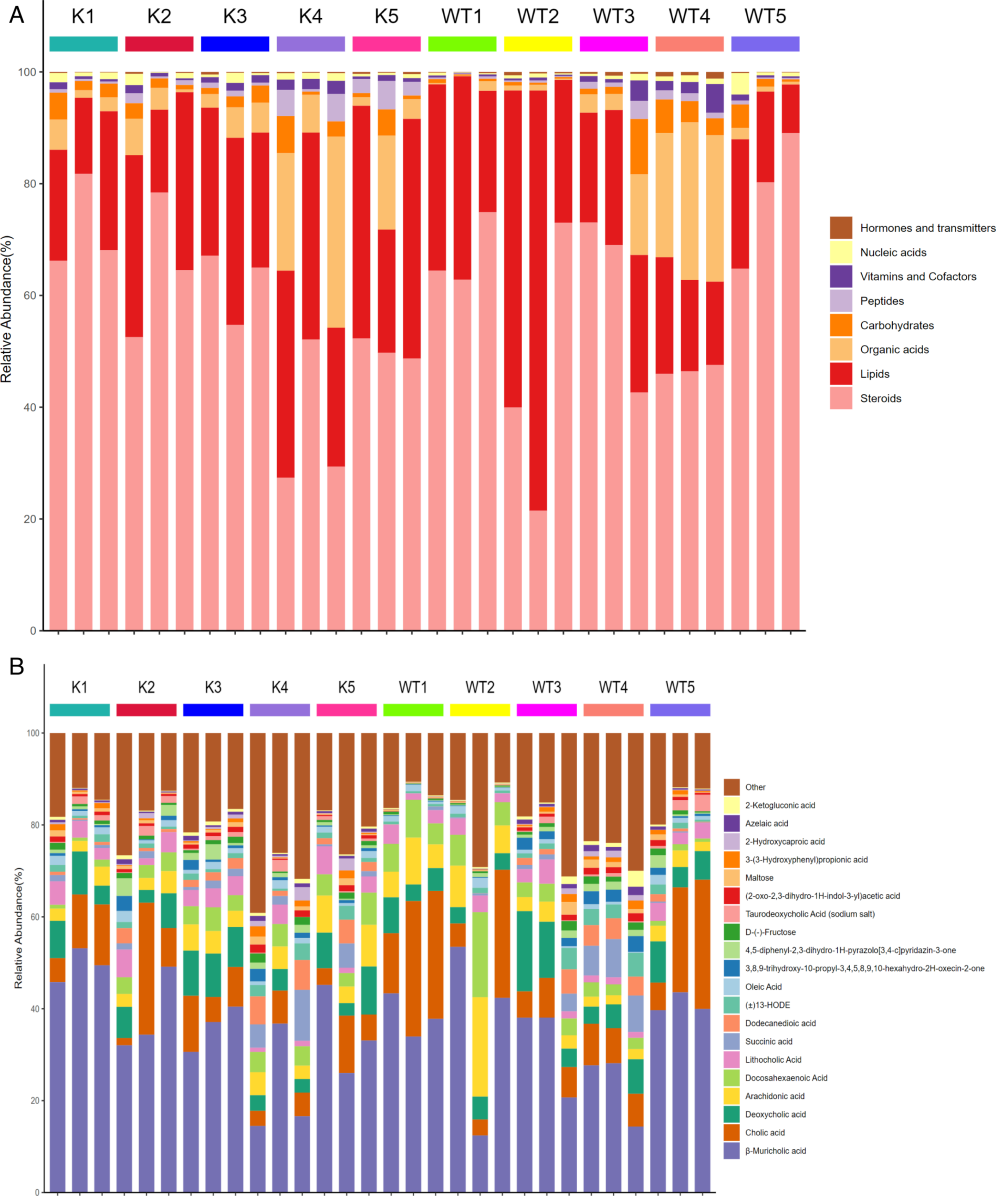
Analysis of intestinal metabolic components. The metabolites of each group of samples are annotated with the KEGG database, and the percentage content of each biological role is calculated (**A**). The content percentage of each metabolite in each sample is calculated, and the differences in metabolite composition and structure between each group are compared (**B**). K, samples from lcn2^−/−^ mice; WT, samples from wild-type mice.

To delve into the relationship between the *Lcn2* gene and gut metabolic components under *M. bovis* infection, we conducted an untargeted metabolomic analysis of gut metabolites in wild-type and Lcn2^−/−^ mice over 1–5 wk postinfection. Notable distinctions emerged, as Lcn2^−/−^ mice, when compared to WT mice, exhibited a decrease in lipid content and a significant increase in organic acids and carbohydrates in the gut during the first and second weeks postinfection. Additionally, steroids content showed a significant decrease in the fifth week postinfection (Fig. 7A). Moreover, we delved into specific metabolic components. The findings highlighted that in comparison to WT mice, Lcn2^−/−^ mice showcased a decrease in cholic acid content in the gut during the first and fifth weeks postinfection. Arachidonic acid content exhibited a decrease in the first and second weeks, whereas β-muricholic acid content significantly increased in the first week postinfection (Fig. 7B).

### The absence of Lcn2 regulates gut metabolic pathways

**Fig 8:**
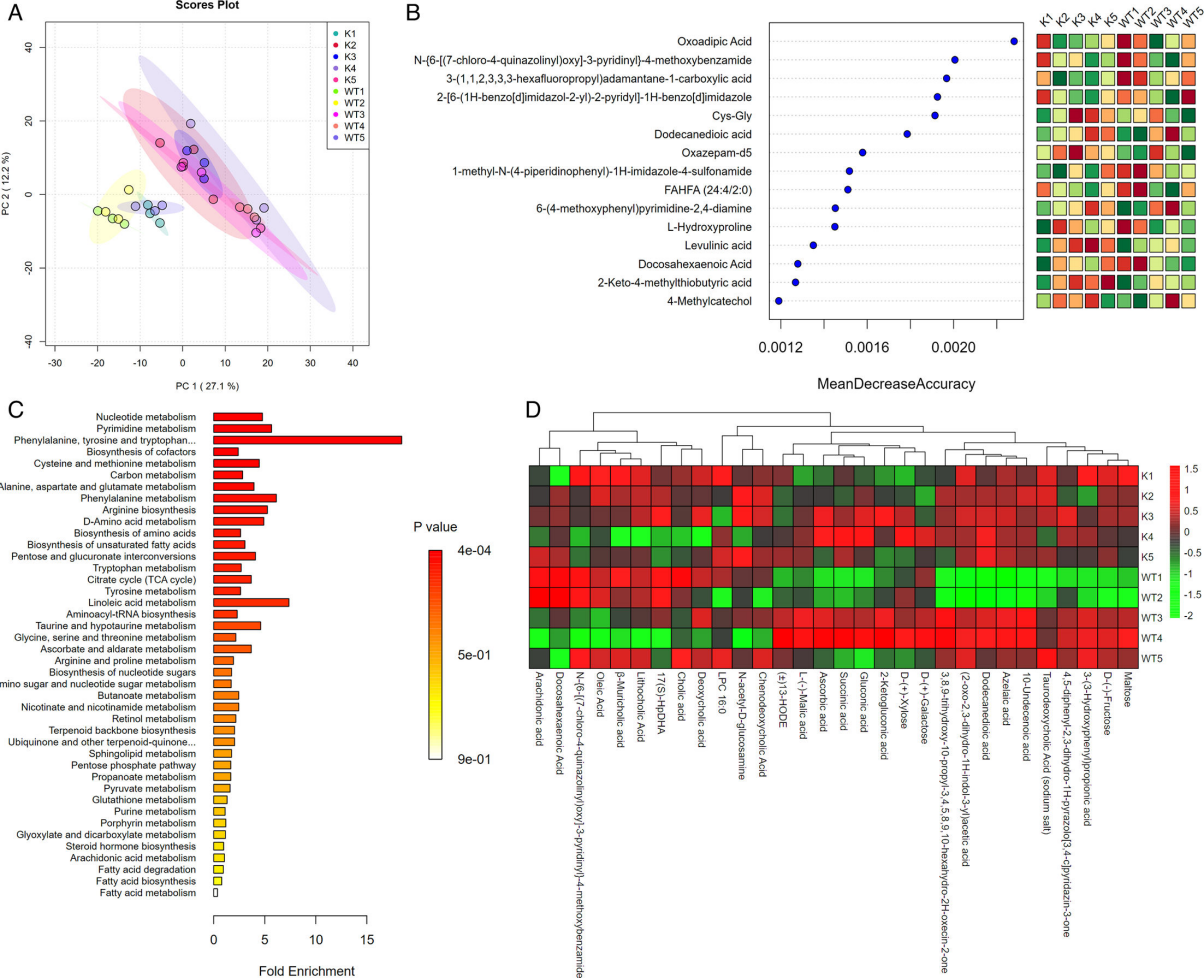
Differential metabolite analysis. (**A**) PCA analysis of metabolites in each group. (**B**) Random forest algorithm analysis. (**C**) Analysis of differential metabolite enrichment metabolic pathways. (**D**) Sample clustering analysis of differential metabolites. K, samples from lcn2^−/−^ mice; WT, samples from wild-type mice.

The PCA results uncovered significant differences in gut metabolites between Lcn2^−/−^ and WT mice post*-M. bovis* infection, illustrating metabolites with substantial disparities (Fig. 8A). The impact of these differential metabolites, such as oxoadipic acid, dodecanedioic acid, and Cys-Gly, on the heterogeneity of observations was further emphasized through random forest analysis, as indicated by their influence across all nodes of the classification tree (Fig. 8B). In the exploration of sample similarities, clustering analysis was employed to construct sample clusters, revealing the distribution of these differential metabolites. In Lcn2^−/−^ mice, notable increases were observed in the levels of taurodeoxycholic acid, 10-undecenoic acid, azelaic acid, dodecanedioic acid, (2-oxo-2,3-dihydro-1H-indol-3-yl) acetic acid, and 3,8,9-trihydroxy-10-propyl-3,4,5,8,9,10-hexahydro-2H-oxecin-2-one (Fig. 8D). Furthermore,Kyoto Encyclopedia of Genes and Genomes (KEGG) pathway enrichment analysis shed light on significant enrichments in nucleotide metabolism, pyrimidine metabolism, and phenylalanine, tyrosine, and tryptophan biosynthesis pathways in Lcn2^−/−^ mice (Fig. 8C).

## DISCUSSION

The infections caused by *M. bovis* in cattle, leading to necrotic pneumonia, mastitis, and arthritis, pose substantial challenges to the livestock industry (22). These diseases not only compromise animal health but also hinder nutritional absorption, adversely affecting cattle growth and the quality of dairy products. Therefore, understanding the physiological impact of *M. bovis* infection on animals is crucial. Lipocalin 2, a protein with a pivotal role in inflammatory responses and tissue damage, has been implicated in microbial invasion responses, suggesting its potential significance in infectious diseases (23). Despite its known role, the specific impact of Lcn2 in *M. bovis* infection, particularly its influence on the gut environment, remains unclear (14). This study aims to explore the role of Lcn2 in the gut environment of mice following *M. bovis* infection, utilizing Lcn2 knockout (Lcn2^−/−^) mice, to assess changes in gut microbiota and metabolic components.

Previous research has demonstrated a direct induction of Lipocalin 2 production in the intestine by the gut microbiota, and mice Lcn2^−/−^ exhibit dysbiosis of the gut microbiome (24). This study found that mice lacking Lcn2 exhibited more severe pulmonary pathological damage following infection with *M. bovis*. Additionally, a decrease in T cell numbers and a reduction in IL-6 levels were observed. These findings suggest that the *Lcn2* gene may regulate the expression of inflammatory factors, thereby modulating the phagocytic activity of immune cells at the site of infection and accelerating the clearance of pathogens. Lcn2 has been found to regulate the activity of dendritic cells and shape immunity to influenza in a microbiome-dependent manner, suggesting its actions are mediated by the microbiome (25). This highlights the potential role of Lcn2 in modulating the diversity of the gut microbiota. In line with previous research, following *M. bovis* infection, Lcn2^−/−^ mice displayed alterations in the number of OTUs of the gut microbiota, indicating a potential crucial role for Lcn2 in maintaining the stability of the gut microbial community. Additionally, significant differences in the α and β diversity of the gut microbiota were observed in Lcn2^−/−^ mice, emphasizing the importance of Lcn2 in the balance of gut microbiota. These changes could have significant implications for the host’s health and disease resistance in the context of *M. bovis* infection.

The intimate link between Lcn2 and gut inflammation associated with microbiome alterations has been established in previous studies. For instance, opportunistic pathogen *Alistipes* spp. exploit siderophores as an iron source, leading to extensive proliferation in Lcn10^−/−^/Il10^−/−^ mice and inducing colitis and right-sided tumors when transferred to Il2^−/−^ mice (26). In the context of this study, specific microbiota, including Akkermansia, Oscillospira, and Bacteroides, were significantly enriched in Lcn2^−/−^ mice, whereas the abundance of Ruminococcus notably decreased. These shifts suggest the potential role of Lcn2 in modulating the composition of the gut microbial community (27). The marked enrichment of *A. muciniphila* is particularly noteworthy due to its positive association with intestinal health and immune regulation. *A. muciniphila* has been shown to enhance the tight junctions of intestinal epithelial cells, thereby reducing the permeation of pathogens and harmful substances (28).

Through its metabolic products, such as short-chain fatty acids, this bacterium can further modulate the intestinal immune response, reinforcing defenses against pathogens (29). In certain bacterial infections like antibiotic-associated colitis, supplementation with *A. muciniphila* has shown promise in restoring a healthy state of the gut microbiome (30). These findings suggest that Lcn2 may influence the host’s health by modulating the abundance of specific microbial communities, providing valuable insights into the role of Lcn2 in the dynamics of the gut microbiome. Additionally, this research may offer clues for the development of novel therapeutic strategies, particularly in the context of gut-related health conditions and microbial dysbiosis.

Alterations in Lcn2 levels have a substantial impact on the abundance of beneficial gut microbiota, consequently influencing the spectrum of gut metabolites, which play a crucial role in the absorption and transport of these metabolites (31). Abnormal expression of Lcn2 in certain disease states, such as inflammatory bowel disease, can disrupt gut metabolism (32). Research indicates that Lcn2 deficiency accelerates the development of gut inflammation and microbiome dysbiosis induced by a short-term high-fat diet (HFD), resulting in a decrease in the production of short-chain fatty acids and the microbes that produce them (33). Furthermore, the absence of Lcn2 has been associated with shifts in the gut microbiome associated with aging toward an unhealthy profile and a reduction in the production of microbial butyrates (34). Lcn2 may also influence the immune response of the gut mucosa, indirectly affecting the gut’s metabolic environment (35). In the context of this study, the absence of Lcn2 significantly impacted the gut metabolic profile, particularly in Lcn2-/- mice, where there was a notable increase in the levels of metabolites such as taurodeoxycholic acid, 10-undecenoic acid, azelaic acid, and dodecanedioic acid. These changes in metabolites may reflect alterations in the gut microenvironment, interacting with shifts in the gut microbiome and thereby affecting intestinal function and the host’s immune response. Thus, the role of Lcn2 in regulating gut metabolic components emerges as a crucial aspect of its function in host defense mechanisms. These findings contribute significant biological insights into the role of Lcn2 in maintaining intestinal health and addressing disease-related processes.

## Data Availability

The raw data reported in this article have been deposited in Nanjing Agricultural University, Nanjing, China (BioProject: PRJNA1033400).
